# Survey data on the impact of COVID-19 on parental engagement across 23 countries

**DOI:** 10.1016/j.dib.2021.106813

**Published:** 2021-02-01

**Authors:** Eliana Maria Osorio-Saez, Nurullah Eryilmaz, Andres Sandoval-Hernandez, Yui-yip Lau, Elma Barahona, Adil Anwar Bhatti, Godfried Caesar Ofoe, Leví Astul Castro Ordóñez, Artemio Arturo Cortez Ochoa, Rafael Ángel Espinoza Pizarro, Esther Fonseca Aguilar, Maria Magdalena Isac, K.V. Dhanapala, Kalyan Kumar Kameshwara, Ysrael Alberto Martínez Contreras, Geberew Tulu Mekonnen, José Fernando Mejía, Catalina Miranda, Shehe Abdalla Moh'd, Ricardo Morales Ulloa, K Kayon Morgan, Thomas Lee Morgan, Sara Mori, Forti Ebenezah Nde, Silvia Panzavolta, Lluís Parcerisa, Carla Leticia Paz, Oscar Picardo, Carolina Piñeros, Pablo Rivera-Vargas, Alessia Rosa, Lina Maria Saldarriaga, Adrián Silveira Aberastury, YM Tang, Kyoko Taniguchi, Ernesto Treviño, Carolina Valladares Celis, Cristóbal Villalobos, Dan Zhao, Allison Zionts

**Affiliations:** a University of Bath, United Kingdom; b The Hong Kong Polytechnic University, Hong Kong; c Universidad Pedagógica Nacional Francisco Morazán, Honduras; d University of Karachi, Pakistan; e Ghana Education Service, Ghana; f University of Bristol, United Kingdom; g Universidad Nacional de Costa Rica, Costa Rica; h KU Leuven, Belgium; i University of Colombo, Sri Lanka; j Pontificia Universidad Católica del Perú, Peru; k University of Tasmania, Australia; l Programa Aulas en Paz - Universidad de los Andes, Colombia; m Pontificia Universidad Católica de Chile, Chile; n State University of Zanzibar, Tanzania; o University of Hartford, The United States; p Sacred Heart University, The United States; q Università Telematica degli Studi (IUL), Italy; r University of Yaounde, Cameroon; s Universitat Autònoma de Barcelona, Spain; t Arizona State University, The United States; u Corporación Colombiana de Padres y Madres - Red PaPaz, Colombia; v Universidad de Barcelona, Spain; w Universidad de la República, Uruguay; x Hiroshima University, Japan; y Goldsmiths, University of London, United Kingdom

**Keywords:** COVID-19, Parental engagement, Acceptance, Confidence, Socioeconomic status, CFA, MG-CFA

## Abstract

This data article describes the dataset of the International COVID-19 Impact on Parental Engagement Study (ICIPES). ICIPES is a collaborative effort of more than 20 institutions to investigate the ways in which, parents and caregivers built capacity engaged with children's learning during the period of social distancing arising from global COVID-19 pandemic. A series of data were collected using an online survey conducted in 23 countries and had a total sample of 4,658 parents/caregivers. The description of the data contained in this article is divided into two main parts. The first part is a descriptive analysis of all the items included in the survey and was performed using tables and figures. The second part refers to the construction of scales. Three scales were constructed and included in the dataset: ‘parental acceptance and confidence in the use of technology’, ‘parental engagement in children's learning’ and ‘socioeconomic status’. The scales were created using Confirmatory Factor Analysis (CFA) and Multi-Group Confirmatory Analysis (MG-CFA) and were adopted to evaluate their cross-cultural comparability (i.e., measurement invariance) across countries and within sub-groups. This dataset will be relevant for researchers in different fields, particularly for those interested in international comparative education.

**Specifications Table**

**Table:** 

Subject	Education, Psychometrics
Specific subject area	Parental Engagement
Type of data	Table, Figure, Text
How data were acquired	Online Survey
Data format	Raw and Analysed Data, Descriptive Statistics
Parameters for data collection	Countries, Location: Area, Parent/carer Gender, Parent/carer Age, Parent/carer years of schooling, Family socioeconomic status, Children's Gender, Children's Age, Children's years of schooling, Number of children in the household, Parental engagement in school activities, Parental use of technology for social purposes, Parental use of technology for building capacity, Parental use of technology tools/resources provided by schools/governments.
Description of data collection	A series of data were collected via online distributed questionnaires in all participating countries (23 countries). The questionnaire was created in an international English version and subsequently translated and adapted to the official languages and localisms of the participating countries. After the first translation, questionnaires were back-translated into English, the equivalence of the questionnaire in the target languages was evaluated and relevant adjustments made. The questionnaires were then distributed through the networks of the participating institutions in each country. The ICIPES target population was parents/caregivers of children between 6 and 16 years old, living with their child and between grade 1 and 13 that represents between 1 and 13 years of schooling, counting from the beginning of Level 1 of the International Standard Classification of Education (ISCED). An intended sample of at least 200 parents was established and countries not reaching this target were flagged. The international English version of the questionnaire can be accessed here: http://dx.doi.org/10.17632/kvvdgvs8zs.2. Due to confidentiality agreements, all details of interviewees’ personal particulars are excluded.
Data source location	Data were collected from 4658 parents/caregivers across 23 countries (Cameroon, Ethiopia, Ghana, Tanzania Zanzibar, China (Mainland, Hong Kong and Macao), Japan, Belgium, Italy, Spain, Turkey, United Kingdom, India, Pakistan, Sri Lanka, Chile, Colombia, Costa Rica, El Salvador, Honduras, Mexico, Peru, Uruguay, The United States) in 5 regions (Africa, East Asia, Europe, South Asia and America).
Data accessibility	Repository name: Mendeley Data Repository: http://dx.doi.org/10.17632/kvvdgvs8zs.2

## Value of the Data

• The database offers first hand valuable information about parental engagement, school support for parents and children, home-schooling and family life balance and parental acceptance and confidence in the use of technology from 23 countries around the world.
• The international database provides a rich environment for examining how parents and caregivers relate to children's learning in this period of social distancing caused by the global COVID-19 outbreak.
• The international database offers data comparable on parental practices during the lockdown across 23 countries and five regions (America, South-Asia, East-Asia, Africa and Europe), allowing investigations on aspects of specific relevance in each of these geographic regions.
• The international dataset contains scales such as parental engagement, parental acceptance and confidence in the use of technology scale and family socioeconomic status, which allow testing hypothesis about the interactions of these and other variables across and within the participating countries.
• The international database involves considerable information for the researchers, analysts, policymakers and education stakeholders to take steps and measures to improve the quality of parental engagement in children's education during and after the lockdown period.

## 1 Data Description

With the advent of the detection of the first case of COVID-19 in the late of November in China and later in the beginning of March in the other countries, an urgent governance step has been initiated by the Ministries of National Education to carry on various educational activities remotely since schools have experienced compulsory shut downs until the end of April-June, depending in which country you are in, to prevent spreading the virus across countries [17]. The pandemic has shown countless barriers that families face daily in their goal of educating their children. It is a unique historical opportunity for researchers and policymakers to understand all the lessons from this global emergency and work closely with parents/caregivers to support them in engaging with children's learning as they are the best partners in mitigating both short and long-term impacts of COVID-19 on children's learning.

Research connects children social and cognitive development to parents' educational practices at home [9]. Mostly, to parental practices that have the potential to provide learning experiences for children, such as: reading to children, using complex language, responsiveness and warmth in interactions and conversations, playing with numbers, painting and drawing, learning about numbers and letters and going to the library [5,^4^,12].

In the current pandemic, parents have spent more time with their children. Moreover, the primary responsibility for enforcing and maintaining young people's educational engagement lies with them. While there is a substantial body of literature which explores parental engagement in education (e.g., [2]), the uniqueness of the current circumstances demands more investigation of how parents are building capacity at home, what activities are they developing with their children, what kind of support they have received from the schools, and how parents have shaped and built their roles and IT skills.

The data provided in this study allows researchers to embark on investigations to the above and other related areas and questions.

### 1.1 Identification variables in the dataset

All ICIPES 2020 data files contain several identification variables that provide information to identify the participants’ important characteristics. The variables do not allow identification of individual parents within countries.

IDCNTRY

This variable indicates the country or participating education system; the data refers to an up to six-digit numeric code based on the ISO 3166 classification, with adaptations reflecting the participating education systems. This variable should always be used as the first linking variable whenever files are linked within and across countries.

CNT

This variable indicates the participant's three-letter alphanumeric code, based on the ISO 3166-1 coding, with adaptations reflecting the participating country.

CNTPARID

This variable indicates the country's three numeric code, based on the ISO 3166–1 coding, plus a unique identifier for each respondent.

REGID

This variable identifies the specific region that each country belongs to. There are five  geographical regions: 1 Africa, 2 East Asia, 3 Europe, 4 South Asia and 5 America.

REG

This variable indicates the participant's three-letter alphanumeric code, based on the ISO 3166- 1 coding, with adaptations reflecting the participating geographical regions.

URN

This variable identifies the specific questionnaire that was administered to each parent. This number was automatically provided by the Online Surveys tool.

In this study, the online survey was conducted with semi-structured questionnaires. Online survey is one of the best ways to reduce the cost when conducting a study, but it is also an effective way to get real data from the online population [13]. A total of 4658 respondents (parents) answered questionnaires from the participating countries: Cameroon, Ethiopia, Ghana, Tanzania, China (i.e., Mainland, Hong Kong, and Macao), Japan, Belgium, Italy, Spain, Turkey, United Kingdom, India, Pakistan, Sri Lanka, Chile, Colombia, Costa Rica, El Salvador, Honduras, Mexico, Peru, Uruguay, the United States. Later, the countries split into five regions: Africa, East Asia, Europe, South Asia, America. Tables 1 to 12 present some characteristics information about countries, regions, and respondents participating in this study.

**Table 1:** Countries participating in ICIPES 2020.

		Operational Codes			
Regions	Countries	Alpha-3	Numeric	Participants(n)	
Africa (AFR)	Cameroon*	CMR	31	10	381
	Ethiopia	ETH	57	171	
	Ghana	GHA	65	142	
	Tanzania	TAZ	172	58	
East Asia (EAS)	China	CHN	36	217	376
	Japan	JPN	35	159	
Europe (EUR)	Belgium*	BEL	16	5	819
	Italy	ITA	83	517	
	Spain*	SPA	164	28	
	Turkey	TUR	179	78	
	United Kingdom	GBR	185	191	
South Asia (SAS)	India	IND	77	54	298
	Pakistan	PAK	131	45	
	Sri Lanka	LKA	165	199	
America (AMR)	Chile	CHL	35	1597	2784
	Colombia	COL	37	94	
	Costa Rica	CRI	40	155	
	El Salvador	SLV	52	83	
	Honduras	HND	74	246	
	Mexico	MEX	110	244	
	Peru*	PER	137	15	
	Uruguay	URY	187	61	
	USA	USA	186	289	
*N=*				4658	4658

⁎ Concerns about the extremely low response rates (less than 10%) for the parents surveys led to a decision not to include the corresponding data in the international database.

**Table 2:** Respondents by Country.

Country	Frequency	Percentage
Chile	1597	34.7
China	217	4.7
Colombia	94	2.0
Costa Rica	155	3.4
El Salvador	83	1.8
Ethiopia	171	3.7
Ghana	142	3.1
Honduras	246	5.3
India	54	1.2
Italy	517	11.2
Japan	159	3.5
Mexico	244	5.3
Pakistan	45	1.0
Sri Lanka	199	4.3
Tanzania&Zanzibar	58	1.3
Turkey	78	1.7
United Kingdom	191	4.2
The United States	289	6.3
Uruguay	61	1.3
Total	4600	100.0

**Table 3:** Respondents by Region.

Region	Frequency	Percentage
Africa	371	8.1
Europe	786	17.1
East Asia	376	8.2
South Asia	298	6.5
America	2769	60.2
Total	4600	100.0

**Table 4:** Respondents by Location.

Location/Area	Frequency	Percentage
Urban	3725	81
Rural	747	16.2
Others	128	2.8
Total	4600	100

**Table 5:** Respondents by Parent/Carer Gender.

Gender	Frequency	Percentage
Mother/Female Guardian	3527	76.67
Father/Male Guardian	1071	23.28
Missing	2	0.04
Total	4600	100

**Table 6:** Respondents by Parent/Carer years of schooling.

Parent/Carer years of schooling	Frequency	Percentage
0 year	13	0.3
1 year	9	0.2
2 year	3	0.1
3 year	17	0.4
4 year	29	0.6
5 year	82	1.8
6 year	57	1.2
7 year	25	0.5
8 year	78	1.7
9 year	39	0.8
10 year	72	1.6
11 year	33	0.7
12 year	203	4.4
13 year	366	8.0
14 year	179	3.9
15 year	800	17.4
16 year	583	12.7
17 year	858	18.7
18 year	336	7.3
19 year	455	9.9
20 year	79	1.7
21 year	20	0.4
22 year	150	3.3
23 year	48	1.0
24 year	7	0.2
Prefer not to say	3	0.1
Missing	56	1.2
Total	4600	100.0

**Table 7:** Respondents by Parent/Carer Age.

Parent/Carer Age	Frequency	Percentage
Under 18 years old	32	0.7
18–24 years old	47	1.0
25–34 years old	740	16.1
35–44 years old	2232	48.5
45–54 years old	1329	28.9
55–64 years old	188	4.1
65–74 years old	30	0.7
75 years or older	2	0.0
Total	4600	100.0

**Table 8:** Respondents by Parent/Carer Main Occupation.

Parent/Carer Main Occupation	Frequency	Percentage
Unemployed, househusband, housewife	509	11.1
91 Elementary trades and related occupations /92 Elementary administration and service occupations	153	3.3
41 Administrative occupations /42 Secretarial and related occupations /61 Caring personal service occupations /62 Leisure, travel and related personal service occupations /63 Community and civil enforcement occupations¹/71 Sales occupations / 72 Customer service occupations / 81 Process, plant and machine operatives / 82 Transport and mobile machine drivers and operatives	747	16.2
12 Other managers and proprietors/ 31 Science, engineering and technology associate professionals / 32 Health and social care associate professionals / Protective service occupations / 34 Culture, media and sports occupations / 35 Business and public service associate professionals / 51 Skilled agricultural and related trades /52 Skilled metal, electrical and electronic trades / 53 Skilled construction and building trades / 54 Textiles, printing and other skilled trades	569	12.4
11 Corporate managers and directors / 21 Science, research, engineering and technology professionals / 22 Health professionals / 23 Teaching and other educational professionals / 24 Business, media and public service professionals	2520	54.8
Missing	102	2.2
Total	4600	100.0

**Table 9:** Parent's Child Gender.

Child gender	Frequency	Percentage
Female	2279	49.5
Male	2303	50.1
Other	18	0.4
Total	4600	100.0

**Table 10:** Parent's Child Age.

Child Age	Frequency	Percentage
6-year-old	691	15.0
7-year-old	470	10.2
8-year-old	464	10.1
9-year-old	392	8.5
10-year-old	448	9.7
11-year-old	388	8.4
12-year-old	402	8.7
13-year-old	307	6.7
14-year-old	303	6.6
15-year-old	264	5.7
16-year-old	411	8.9
Missing	60	1.3
Total	4600	100.0

**Table 11:** Parent's child years of schooling.

Child years of schooling	Frequency	Percentage
Pre-school	237	5.2
1	479	10.4
2	516	11.2
3	458	10.0
4	414	9.0
5	464	10.1
6	365	7.9
7	417	9.1
8	352	7.7
9	273	5.9
10	251	5.5
11	178	3.9
12	50	1.1
13	18	0.4
14	1	0.0
Missing	127	2.8
Total	4600	100.0

**Table 12:** Children in the household.

How many siblings living in the same household?	Frequency	Percentage
0	1482	32.2
1	1676	36.4
2	787	17.1
3	223	4.8
4	214	4.7
5	118	2.6
6	50	1.1
7	47	1.0
8	2	0.0
9		
10	1	0.0
Total	4600	100.0

The following section provides information about the procedure followed to construct three scales in ICIPES 2020.

Social cognitive learning theory [3] and the theory of acceptance and use of technology [14], [15], [16],1] formed the conceptual framework for these scales. The social cognitive learning theory provides a socially appropriate framework for understanding how parents learn to deal with technology at home from their observations and interactions with other parents, teachers and their children. The second explains the factors associated with parental acceptance and confidence in the use of technology.

Before constructing the three scales, we constructed and implemented normalised weights (also known as senate weights) (SENWT in the dataset) to make sure that when constructing these three scales, all countries are represented equally regardless of their sample sizes. SENWT can also be used when analysing the pooled sample (all countries) to ensure the equal contribution of each country to the results.

### 1.2 Variables

#### 1.2.1 Parental engagement

The parental engagement scale was constructed using the following questions: Q21_2, Q21_3, Q22_2, Q22_3, and Q22_6 from the data set.

Always, Often, Occasionally, Rarely, Never (from 0 to 4)

• Q21_2 Follow my ideas about what my children need to learn
• Q21_3 Mix my own ideas with the school's plan on what my children need to learn
• Q22_2 I list and prepare the activities myself before developing them with my child(ren)
• Q22_3 My children and I have a set home-schooling timetable.
• Q22_6 I develop with my children spontaneous learning activities not necessarily school-related such as cooking, woodwork, online games, physical activities, etc.

#### 1.2.2 Socioeconomic status (SES)

Socioeconomic status (SES) was constructed using the following questions: Q5, Q7, Q13N, and Q14.

Q5 What do you do in your main job? (e.g., teach high school students, help the cook prepare meals in a restaurant, manage a sales team). This was an open question that was recorded into an ordinal variable following the list of occupations described in the one-digit ISCO (International Standard Classification of Occupations).

Q7 In a normal month, what is your total household income? This variable was recorded by grouping the income level reported in deciles of income within each country.

Q13N is composed of How many usable devices are there in the house? (Smartphones, tablets or iPads, laptops, desktops).

Q14 How many computers per child have you got at home?

#### 1.2.3 Parental acceptance and confidence in the use of technology

Parental engagement scale was constructed as a second-order construct, with constructs measuring the parents’ level of parental acceptance and confidence in the use of technology as ‘tools’, ‘for social purposes’ and ‘self- perceived capacity’. The items asked parents about the frequency with which they carry out different activities using technology (response options: Always, Often, Occasionally, Rarely Never), and how confident they felt carrying out these activities (response options: Not at all confident, Slightly confident, Moderately confident, Quite confident, Extremely confident).

Parental acceptance and confidence in the use of technology= tool + social + capacity.

• tool=Q22_1 + Q24_1 + Q24_5;
• social=Q21_4 + Q21_5 + Q21_6 + Q24_12;
• capacity=Q24_2 + Q24_3 +Q24_4 + Q24_6 + Q24_7 + Q24_8 + Q24_9 + Q24_10+Q24_11+ Q21_7

### 1.3 Analytical strategy

#### 1.3.1 Confirmatory factor analysis (CFA)

Confirmatory Factor Analysis (CFA) was used to estimate the model for the three scales and for each country using maximum likelihood (ML). Missing data was handled with listwise deletion. Model fit was evaluated using the Comparative Fit Index (CFI) and the Tucker-Lewis Index (TLI) as the goodness of fit statistics, and the root-mean-squared error of approximation (RMSEA) and the standardized root mean squared residual (SRMR) as residual fit statistics. Acceptable model fit was guided by the cut-offs (CFI > 0.90; TLI > 0.90; RMSEA < 0.10; and SRMR < 0.08) as suggested by [8].

Internal Consistency

After constructing three scales, in order to evaluate reliability (internal consistency), we used Cronbach's alpha coefficient [6].

Multi-Group Confirmatory Factor Analysis (MG-CFA)

In order to evaluate the extent to which the scales can be validly compared across countries and geographical areas, we ran Multi-Group Confirmatory Factor Analysis (MG-CFA) first for the pooled sample including all participating countries, and later for countries within sub-groups (America, South Asia, East Asia, Africa and Europe) [10]. Here, we adopted the same strategy as [11] and [7] to conduct analysis and to interpret the results (for more information about procedure see these two papers [11] and [7]).

### 1.4 Important information for potential users

The following tables include important information for potential users to be able to interpret the scales correctly.

#### 1.4.1 Parental engagement scale

**Fig. 1:**
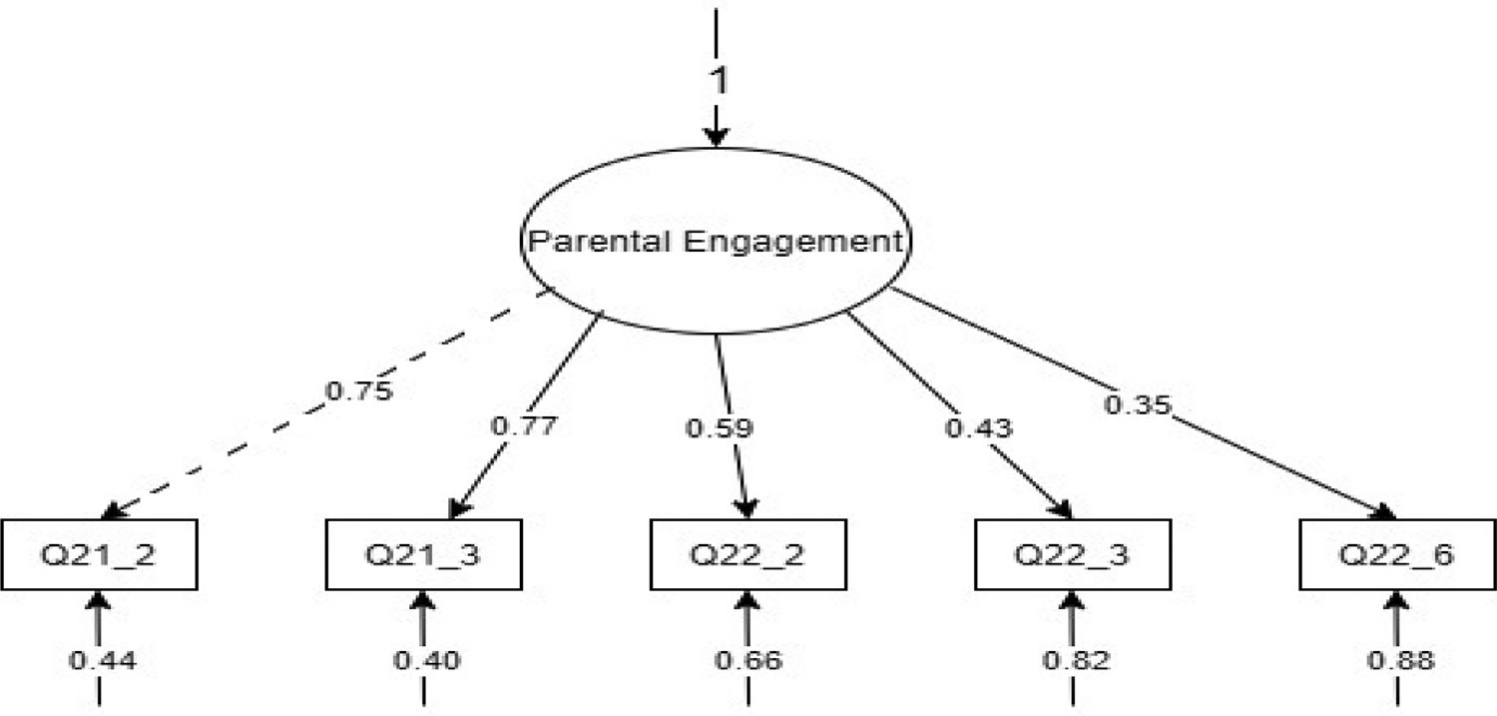
Measurement model for Parental Engagement.

Tables 13 and 14, Fig. 1

**Table 13:** Confirmatory Factor Analysis Model Fit for engagement scale for all countries.

Fit statistics	Chi-square	df	CFI	TLI	RMSEA	SRMR	Reliability
Engagement(*n* = 4657)	508.122	5	0.898	0.796	0.147	0.056	0.7

Note. df = degree of freedom; CFI = Comparative Fit index; TLI = Tucker-Lewis index; RMSEA = Root Mean Square Error of Approximation; SRMR = Standardized Root Mean Square Residual.

**Table 14:** Confirmatory factor analysis model for engagement scale for each country.

Educational System	Reliability	CFI	TLI	RMSEA	SRMR	Degrees of freedom	Test statistics	n
Ethiopia(57)	0.8	0.889	0.779	0.188	0.055	5	35.216	171
Ghana(65)	0.74	0.945	0.889	0.106	0.044	5	12.917	142
Tanzania(172)	0.79	1	1.087	0	0.026	5	2.068	58
China(36)	0.82	0.946	0.892	0.131	0.039	5	23.663	217
Japan(85)	0.7	0.905	0.809	0.135	0.057	5	19.563	159
Italy(83)	0.75	0.954	0.907	0.112	0.044	5	37.611	517
Turkey(179)	0.78	0.884	0.767	0.195	0.069	5	19.774	78
UK(185)	0.74	0.911	0.821	0.141	0.052	5	23.936	191
India(77)	0.71	1	1.183	0	0.031	5	2.02	53
Pakistan(131)	0.84	1	1.004	0	0.05	5	4.791	45
SriLanka(165)	0.8	0.948	0.895	0.129	0.037	5	21.491	199
Chile(35)	0.67	0.869	0.738	0.153	0.072	5	192.119	1597
Colombia(37)	0.5	0.935	0.871	0.073	0.057	5	7.529	94
Costarica(40)	0.69	0.892	0.783	0.142	0.065	5	20.521	155
ElSalvador(52)	0.73	0.852	0.704	0.218	0.098	5	24.72	83
Honduras(74)	0.68	0.707	0.414	0.244	0.113	5	78.059	246
Mexico(110)	0.63	0.762	0.524	0.227	0.101	5	67.954	244
Uruguay(187)	0.65	1	1.018	0	0.05	5	4.607	61
USA(186)	0.73	0.987	0.973	0.049	0.025	5	8.504	289

Note. df = degree of freedom; CFI = Comparative Fit index; TLI = Tucker-Lewis index; RMSEA = Root Mean Square Error of Approximation; SRMR = Standardized Root Mean Square Residual.

#### 1.4.2 MG-CFA result for parental engagement scale

Table 15, Table 16, Table 17, Table 18, Table 19, Table 20

**Table 15:** Confirmatory Factor Analysis for all countries for engagement scale.

Model	Chi-Square	df	RMSEA	SRMR	CFI	TLI	Change (CFI)
All groups	581.5424	5	0.157354	0.058959	0.891463	0.782926	
**Configural invariance**	**607.0634**	**95**	**0.149226**	**0.06181**	**0.898439**	**0.796879**	
Metric invariance	1126.971	167	0.154105	0.106691	0.809603	0.783381	−0.08884
Scalar invariance	1986.75	239	0.173814	0.13809	0.653358	0.724427	−0.15625
Strict invariance	2365.486	329	0.159915	0.153451	0.596091	0.76674	−0.05727

**Table 16:** Confirmatory Factor Analysis for Africa for engagement scale.

							371(4)
Model	Chi-Square	df	RMSEA	SRMR	CFI	TLI	Change (CFI)
All groups	20.17588	5	0.090449	0.029731	0.968512	0.937024	
Configural invariance	50.20182	15	0.137756	0.04603	0.927239	0.854477	
Metric invariance	55.42492	23	0.10677	0.057237	0.932978	0.91258	0.00574
**Scalar invariance**	**67.30136**	**31**	**0.097309**	**0.065941**	**0.924966**	**0.927386**	**−0.00801**
Strict invariance	96.38475	41	0.104515	0.085205	0.885521	0.916235	−0.03945

**Table 17:** Confirmatory Factor Analysis for Europe for engagement scale.

							786(4)
Model	Chi-Square	df	RMSEA	SRMR	CFI	TLI	Change (CFI)
All groups	98.14603	5	0.153952	0.050065	0.910994	0.821987	
**Configural invariance**	**81.32066**	**15**	**0.129906**	**0.048239**	**0.936394**	**0.872788**	
Metric invariance	145.07	23	0.142328	0.075245	0.882927	0.847296	−0.053467279
Scalar invariance	197.5008	31	0.143178	0.091356	0.840315	0.845466	−0.042612133
Strict invariance	207.5371	41	0.124513	0.089347	0.84028	0.883132

**Table 18:** Confirmatory Factor Analysis for East Asia for engagement scale.

							376(3)
Model	Chi-Square	df	RMSEA	SRMR	CFI	TLI	Change (CFI)
All groups	46.41285	5	0.148419	0.051417	0.917672	0.835344	
Configural invariance	43.22629	10	0.132942	0.046728	0.933142	0.866284	
**Metric invariance**	**54.86896**	**14**	**0.12461**	**0.071612**	**0.917763**	**0.882519**	**−0.015378604**
Scalar invariance	112.9605	18	0.167516	0.115977	0.80892	0.787689	−0.108843147
Strict invariance	148.875	23	0.170619	0.128463	0.746714	0.779751	−0.062206288

**Table 19:** Confirmatory Factor Analysis for south Asia for engagement scale.

							279(3)
Model	Chi-Square	df	RMSEA	SRMR	CFI	TLI	Change (CFI)
All groups	27.89402	5	0.124165	0.037326	0.95517	0.910341	
Configural invariance	28.30219	15	0.094645	0.037787	0.97069	0.941379	
**Metric invariance**	**54.36807**	**23**	**0.117371**	**0.084387**	**0.930883**	**0.909847**	**−0.039806715**
Scalar invariance	86.92192	31	0.134987	0.098635	0.876781	0.880755	−0.054102414
Strict invariance	114.4042	41	0.134478	0.102205	0.83826	0.881653	−0.038520836

**Table 20:** Confirmatory Factor Analysis for America for engagement scale.

							2769(9)
Model	Chi-Square	df	RMSEA	SRMR	CFI	TLI	Change (CFI)
All groups	359.2043	5	0.159949	0.071789	0.861349	0.722699	
Configural invariance	404.0125	40	0.162148	0.072401	0.858066	0.716132	
**Metric invariance**	**496.4607**	**68**	**0.134923**	**0.087906**	**0.832937**	**0.803455**	**−0.025129336**
Scalar invariance	670.0427	96	0.131438	0.09858	0.776172	0.813477	−0.056764687
Strict invariance	749.5524	131	0.116798	0.10514	0.758817	0.852713	−0.017355009

#### 1.4.3 Socioeconomic status scale

**Fig. 2:**
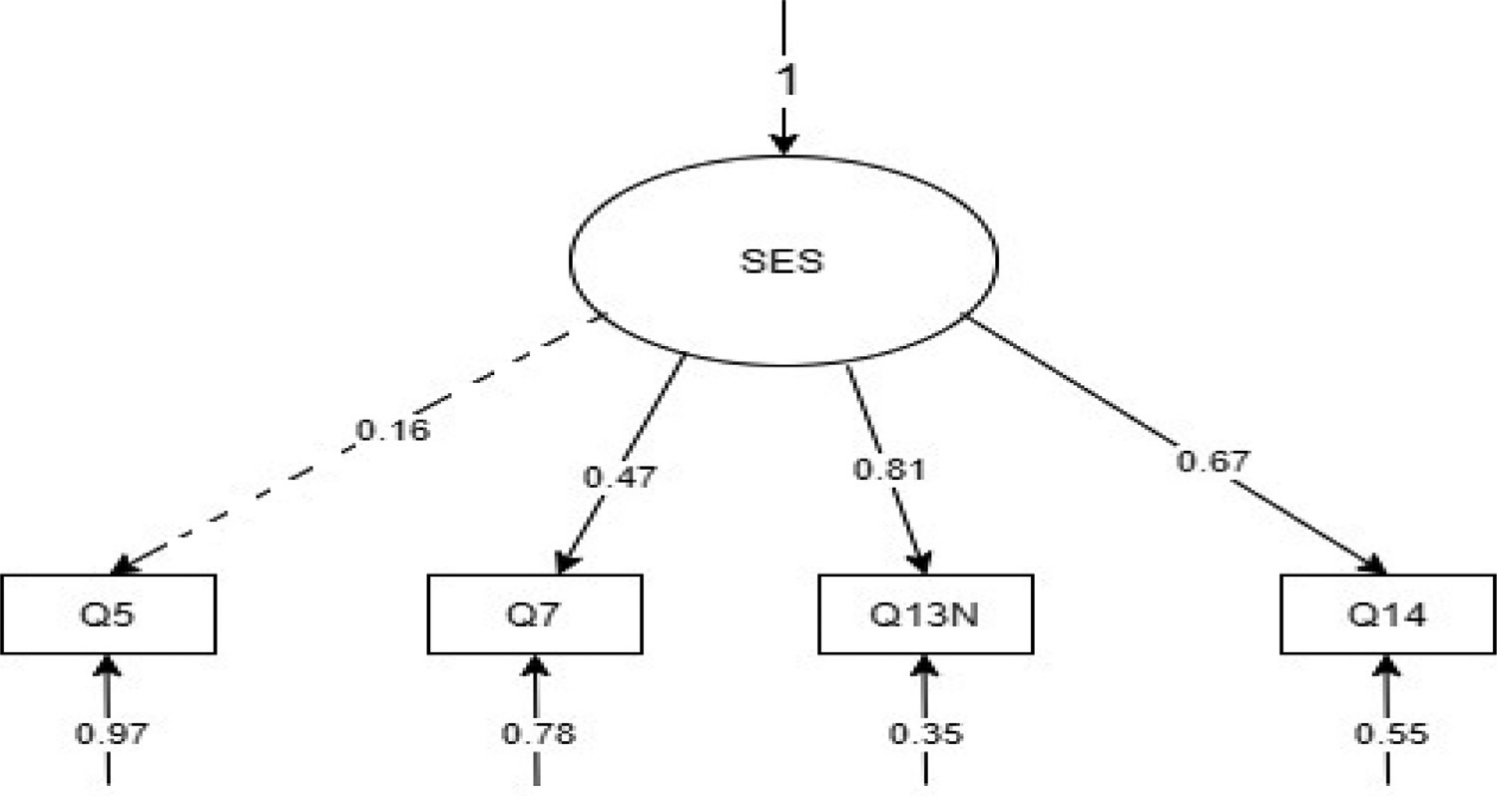
Measurement model for Socioeconomic status.

Tables 21 and 22, Fig. 2

**Table 21:** Confirmatory Factor Analysis Model Fit for SES scale for all countries.

Fit statistics	Chi-square	df	CFI	TLI	RMSEA	SRMR	Reliability
SES(*n* = 4136)	19.388	2	0.992	0.977	0.046	0.015	0.62

Note. df = degree of freedom; CFI = Comparative Fit index; TLI = Tucker-Lewis index; RMSEA = Root Mean Square Error of Approximation; SRMR = Standardized Root Mean Square Residual.

**Table 22:** Confirmatory factor analysis model for SES scale for each country.

Educational system	Reliability	CFI	TLI	RMSEA	SRMR	Degrees of freedom	Test statistics	n
Ethiopia(57)	0.5	1	1.055	0	0.013	2	0.443	169
Ghana(65)	0.44	0.979	0.938	0.059	0.04	2	2.751	108
Tanzania(172)	0.51	0.771	0.312	0.181	0.068	2	5.423	52
China(36)	0.46	0.812	0.435	0.154	0.054	2	9.834	166
Japan(85)	0.46	0.862	0.586	0.139	0.056	2	7.617	145
Italy(83)	0.61	0.949	0.848	0.107	0.035	2	12.271	450
Turkey(179)	0.55	1	1.012	0	0.042	2	1.891	78
UK(185)	0.5	0.942	0.827	0.104	0.044	2	5.24	158
India(77)	0.61	0.98	0.939	0.069	0.049	2	2.509	54
Pakistan(131)	0.55	0.87	0.61	0.205	0.09	2	5.037	36
SriLanka(165)	0.69	0.997	0.991	0.029	0.021	2	2.33	199
Chile(35)	0.65	0.839	0.518	0.224	0.072	2	162.338	1597
Colombia(37)	0.7	0.934	0.803	0.18	0.051	2	7.482	85
Costarica(40)	0.81	0.995	0.984	0.06	0.02	2	3.036	143
Elsalvador(52)	0.75	1	1.085	0	0.006	2	0.075	71
Honduras(74)	0.57	0.99	0.969	0.047	0.025	2	2.981	223
Mexico(110)	0.74	0.987	0.96	0.082	0.024	2	4.787	206
Uruguay(187)	0.59	0.992	0.975	0.047	0.046	2	2.254	58
USA(186)								

Note. df = degree of freedom; CFI = Comparative Fit index; TLI = Tucker-Lewis index; RMSEA = Root Mean Square Error of Approximation; SRMR = Standardized Root Mean Square Residual.

#### 1.4.4 MG-CFA result for socioeconomic status scale

Table 23, Table 24, Table 25, Table 26, Table 27, Table 28

**Table 23:** Confirmatory Factor Analysis for all countries for SES scale.

Model	Chi-Square	df	RMSEA	SRMR	CFI	TLI	Change (CFI)
All groups	19.38766	2	0.045847	0.015055	0.992326	0.976977	
**Configural invariance**	**1233.791**	**308**	**0.125733**	**0.065177**	**0.827353**	**0.74103**	
Metric invariance	1747.675	434	0.126173	0.096277	0.755019	0.739214	−0.07233
Scalar invariance	5079.804	560	0.206031	0.281879	0.157122	0.304626	−0.5979
Strict invariance	7739.431	707	0.228723	0.401474	0	0.143015	−0.15712

**Table 24:** Confirmatory Factor Analysis for Africa countries for SES scale.

Model	Chi-Square	df	RMSEA	SRMR	CFI	TLI	Change (CFI)
All groups	1.754282	2	0	0.014459	1	1.005694	
Configural invariance	8.616476	6	0.063059	0.030384	0.980809	0.942426	
**Metric invariance**	**13.11633**	**12**	**0.029125**	**0.044798**	**0.991812**	**0.987718**	**0.011003**
Scalar invariance	62.44294	18	0.150047	0.114559	0.674022	0.674022	−0.31779
Strict invariance	90.97313	26	0.150953	0.172059	0.523439	0.670073	−0.15058

**Table 25:** Confirmatory Factor Analysis for Europe countries for SES scale.

Model	Chi-Square	df	RMSEA	SRMR	CFI	TLI	Change (CFI)
All groups	55.70191	2	0.202624	0.059631	0.802704	0.408112	
**Configural invariance**	**198.7235**	**56**	**0.122532**	**0.060901**	**0.805383**	**0.708075**	
Metric invariance	256.2546	74	0.120453	0.080512	0.751479	0.717895	−0.0539
Scalar invariance	339.5859	92	0.125911	0.095679	0.662394	0.691751	−0.08909
Strict invariance	496.084	113	0.14132	0.133366	0.477629	0.611689	−0.18476

**Table 26:** Confirmatory Factor Analysis for East Asia countries for SES scale.

Model	Chi-Square	df	RMSEA	SRMR	CFI	TLI	Change (CFI)
All groups	98.42404	14	0.139248	0.084757	0.654562	0.481844	
Configural invariance	17.45187	4	0.147061	0.054767	0.83665	0.50995	
**Metric invariance**	**20.10864**	**7**	**0.10974**	**0.061244**	**0.840818**	**0.727117**	**0.004168**
Scalar invariance	67.61635	10	0.19249	0.12439	0.300348	0.160417	−0.54047
Strict invariance	76.321	14	0.169195	0.142274	0.243218	0.35133	−0.05713

**Table 27:** Confirmatory Factor Analysis for South Asia countries for SES scale.

Model	Chi-Square	df	RMSEA	SRMR	CFI	TLI	Change (CFI)
All groups	2.259465	2	0.023596	0.021439	0.997472	0.992416	
**Configural invariance**	**8.716322**	**6**	**0.076348**	**0.033266**	**0.979696**	**0.939088**	
Metric invariance	25.51962	12	0.120441	0.070277	0.898943	0.848415	−0.08075
Scalar invariance	52.17098	18	0.156342	0.100213	0.744578	0.744578	−0.15437
Strict invariance	115.2477	26	0.21023	0.220021	0.332889	0.538154	−0.41169

**Table 28:** Confirmatory Factor Analysis for America countries for SES scale.

Model	Chi-Square	df	RMSEA	SRMR	CFI	TLI	Change (CFI)
All groups	55.52348	2	0.101279	0.030157	0.963561	0.890683	
**Configural invariance**	**184.13**	**16**	**0.179503**	**0.050682**	**0.898509**	**0.695528**	
Metric invariance	277.5462	37	0.141191	0.074167	0.854796	0.811627	−0.04371
Scalar invariance	883.7312	58	0.208936	0.139707	0.501553	0.587492	−0.35324
Strict invariance	2221.39	86	0.275929	0.295485	0	0.28055	−0.50155

#### 1.4.5 Acceptance and confidence scale

**Fig. 3:**
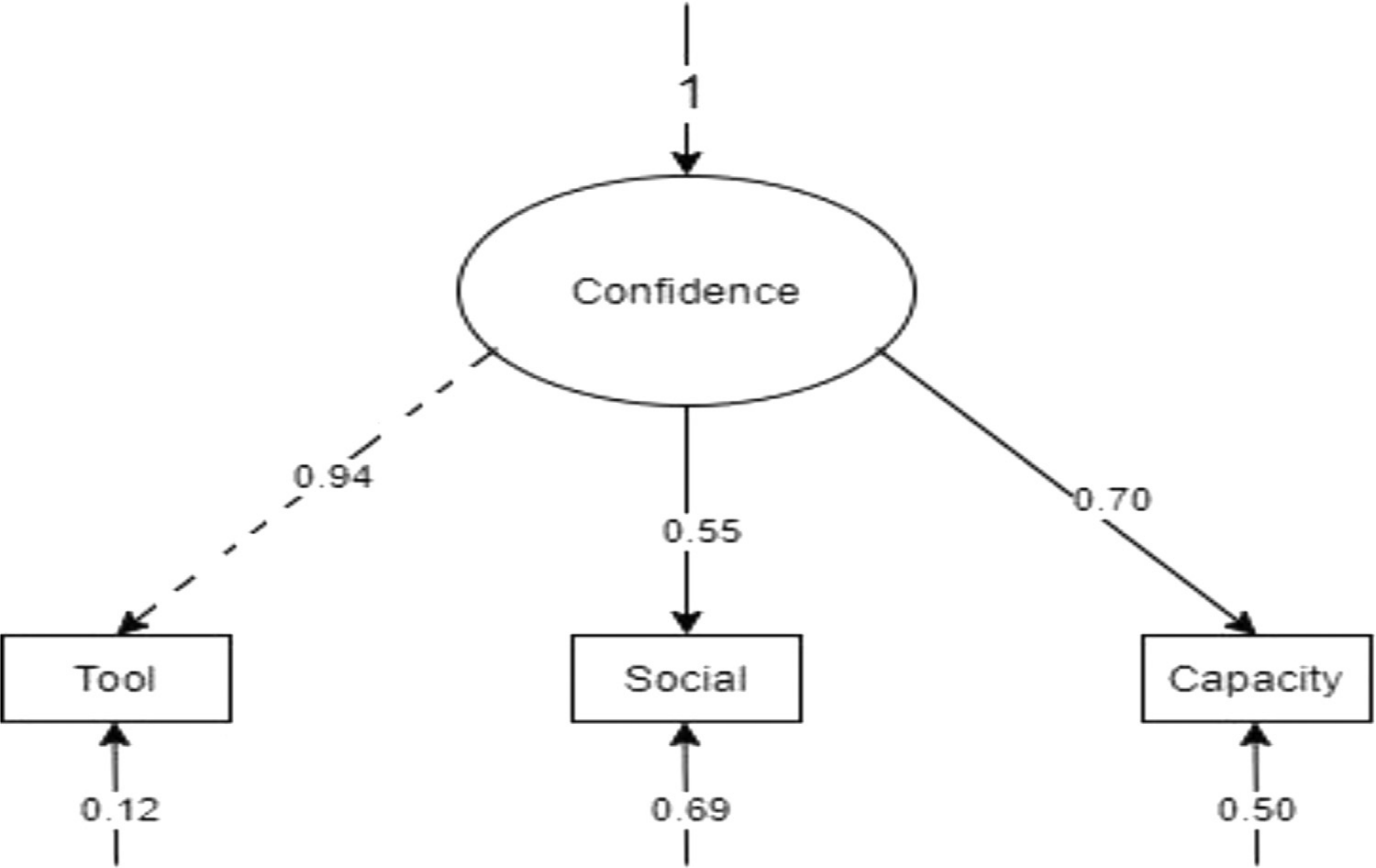
Measurement model for acceptance and confidence scale.

Tables 29 and 30, Fig. 3

**Table 29:** Confirmatory Factor Analysis Model Fit for acceptance and confidence scale for all countries.

Fit statistics	Chi-square	df	CFI	TLI	RMSEA	SRMR	reliability
acceptance(*n* = 4642)	0	0	1	1	0	0	0.78

Note. df = degree of freedom; CFI = Comparative Fit index; TLI = Tucker-Lewis index; RMSEA = Root Mean Square Error of Approximation; SRMR = Standardized Root Mean Square Residual.

**Table 30:** Standardized factor loadings and intercepts for acceptance and confidence scale for each country.

		Factor loadings			Intercepts			
Educational system	Reliability	Tool	Social	Capacity	Tool	Social	Capacity	n
Ethiopia(57)	0.7	0.95	0.339	0.759	2.085	2.588	2.063	171
Ghana(65)	0.57	1.932	0.142	0.311	1.603	2.174	1.775	142
Tanzania(172)	0.69	0.761	0.342	0.916	1.822	2.134	2.373	58
China(36)	0.76	0.789	0.494	0.904	3.104	2.637	2.712	217
Japan(85)	0.74	0.701	0.505	0.91	2.651	4.798	2.456	159
Italy(83)	0.77	0.875	0.58	0.744	3.081	3.948	3.006	517
Turkey(179)	0.79	0.874	0.555	0.827	2.431	1.993	2.059	78
UK(185)	0.78	0.898	0.617	0.719	3.581	3.519	3.52	191
India(77)	0.84	0.928	0.681	0.839	2.173	1.907	2.28	48
Pakistan(131)	0.8	0.714	0.753	0.894	1.827	1.513	1.431	45
SriLanka(165)	0.81	0.921	0.542	0.851	2.148	2.285	2.129	199
Chile(35)	0.74	0.857	0.513	0.737	3.554	3.576	3.301	1597
Colombia(37)	0.73	0.98	0.424	0.711	3.032	3.628	2.811	94
Costarica(40)	0.77	0.965	0.517	0.721	2.785	3.118	2.622	155
Elsalvador(52)	0.76	0.793	0.561	0.807	3.599	3.053	3.551	83
Honduras(74)	0.69	0.734	0.465	0.773	3.245	3.429	2.79	246
Mexico(110)	0.82	0.851	0.614	0.891	2.573	3.002	2.725	244
Uruguay(187)	0.66	0.854	0.391	0.682	4.068	4.699	3.232	61
USA(186)	0.75	0.966	0.482	0.723	2.882	2.509	3.225	289

#### 1.4.6 MG-CFA result for acceptance and confidence scale

Table 31, Table 32, Table 33, Table 34, Table 35, Table 36

**Table 31:** Confirmatory Factor Analysis for all countries for acceptance scale.

Model	Chi-Square	df	RMSEA	SRMR	CFI	TLI	Change (CFI)
All groups	0	0	0	0	1	1	
Configural invariance	0	0	0	0	1	1	
**Metric invariance**	**85.40701865**	**36**	**0.075422**	**0.038111542**	**0.987113**	**0.979595**	**−0.01289**
Scalar invariance	644.5433347	72	0.181548	0.096383494	0.850658	0.881771	−0.13645
Strict invariance	899.9196701	126	0.159557	0.123705734	0.798131	0.908678	−0.05253

**Table 32:** Confirmatory Factor Analysis for Africa for acceptance and confidence scale.

							371(3)
Model	Chi-Square	df	RMSEA	SRMR	CFI	TLI	Change (CFI)
All groups	0	0	0	0	1	1	
Configural invariance	0	0	0	0	1	1	
Metric invariance	6.500133592	4	0.071093	0.036222553	0.990338	0.97826	−0.00966
Scalar invariance	28.55747519	8	0.14415	0.077308907	0.92055	0.910619	−0.06979
**Strict invariance**	**39.78122672**	**14**	**0.122029**	**0.095684283**	**0.900362**	**0.935947**	**−0.02019**

**Table 33:** Confirmatory Factor Analysis for Europe for acceptance and confidence scale.

							786(3)
Model	Chi-Square	df	RMSEA	SRMR	CFI	TLI	Change (CFI)
All groups	0	0	0	0	1	1	
Configural invariance	0	0	0	0	1	1	
**Metric invariance**	**4.743893007**	**4**	**0.026642**	**0.020968118**	**0.998937**	**0.997609**	**−0.00106**
Scalar invariance	92.85405382	8	0.201206	0.088004477	0.878763	0.863608	−0.12017
Strict invariance	142.4050703	14	0.187101	0.120830664	0.816538	0.88206	−0.06222

**Table 34:** Confirmatory Factor Analysis for East Asia for acceptance and confidence scale.

							376(3)
Model	Chi-Square	df	RMSEA	SRMR	CFI	TLI	Change (CFI)
All groups	0	0	0	0	1	1	
Configural invariance	0	0	0	0	1	1	
**Metric invariance**	**2.552421949**	**2**	**0.03833**	**0.034734358**	**0.998282**	**0.994846**	**−0.00172**
Scalar invariance	98.42126974	4	0.354345	0.172117284	0.706356	0.559535	−0.29193
Strict invariance	124.9045583	7	0.299321	0.233652999	0.633325	0.685707	−0.07303

**Table 35:** Confirmatory Factor Analysis for south Asia for acceptance and confidence  scale.

							279(3)
Model	Chi-Square	df	RMSEA	SRMR	CFI	TLI	Change (CFI)
All groups	0	0	0	0	1	1	
Configural invariance	0	0	0	0	1	1	
Metric invariance	6.521818998	4	0.081896	0.047729416	0.992846	0.983904	−0.00715
**Scalar invariance**	**34.89431204**	**8**	**0.189113**	**0.081810798**	**0.923706**	**0.914169**	**−0.06914**
Strict invariance	52.57001258	14	0.171197	0.081061567	0.890584	0.929661	−0.03312

**Table 36:** Confirmatory Factor Analysis for America for acceptance and confidence scale.

							2769(9)
Model	Chi-Square	df	RMSEA	SRMR	CFI	TLI	Change (CFI)
All groups	0	0	0	0	1	1	
Configural invariance	0	0	0	0	1	1	
Metric invariance	18.70874018	14	0.031173	0.019873832	0.997861	0.996333	−0.00214
Scalar invariance	191.561156	28	0.129911	0.056235716	0.92569	0.936306	−0.07217
**Strict invariance**	**242.2002961**	**49**	**0.106731**	**0.067470981**	**0.912224**	**0.957008**	**−0.01347**

## 2 Experimental Design, Materials and Design

The researchers employed an online survey research design to gather data from 2658 respondents from 23 countries all over the world. All countries are Cameroon, Ethiopia, Ghana, Tanzania Zanzibar, China (Mainland, Hong Kong and Macao), Japan, Belgium, Italy, Spain, Turkey, United Kingdom, India, Pakistan, Sri Lanka, Chile, Colombia, Costa Rica, El Salvador, Honduras, Mexico, Peru, Uruguay and the United States. The countries then divided into five regions which are Africa, East Asia, Europe, South Asia and America. Data were obtained using a semi-structured questionnaire (Appendix). The questionnaire consists of several sections. Section 1 and 2 gathered information about the parents and their child. Section 3 gathered information about the children's school and their access to the internet. Section 4 gathered information about the COVID 19 impact in terms of parents’ new role at home. Section 5 gathered information about teaching ideas and practices in terms of home-schooling. The first part is a descriptive analysis of all the items included in the survey and was performed using tables ( see, descriptive part, Tables 1 to 12). The second part refers to the construction of scales (see variables part). Three scales were constructed and included in the dataset: ‘parental acceptance and confidence in the use of technology’, ‘parental engagement in children's learning’ and ‘socioeconomic status’. The scales were created using Confirmatory Factor Analysis (CFA) and Multi-Group Confirmatory Analysis (MG-CFA) was adopted to evaluate their cross-cultural comparability (i.e., measurement invariance) across countries and within sub-groups. All analyses are executed in the R statistical software (R Core Team, 2019), installing *lavaan* and *lavaan.survey* packages developed by Rosseel (2012) and Oberski (2014), respectively.

## Ethics Statement

Informed consent was obtained from all individual participants included in the data collection process. The research ethics committee of the University of Bath provided ethical approval EIRA1–5408.

## CRediT Author Statement

**Eliana Maria Osorio-Saez and Andres Sandoval-Hernandez:** Conceptualization and Methodology; **Nurullah Eryilmaz:** Data curation and Data Analysis; **Nurullah Eryilmaz and Eliana Maria Osorio-Saez:** Writing- Original draft preparation; **Andres Sandoval-Hernandez:** Supervision; **Yui-yip Lau:** Reviewing and Editing; **Eliana Maria Osorio-Saez, Nurullah Eryilmaz, Andres Sandoval-Hernandez, Yui-yip Lau, Elma Barahona, Adil Anwar Bhatti, Godfried Ofoe Caesar, Leví Astul Castro Ordóñez, Artemio Arturo Cortez Ochoa, Rafael Ángel Espinoza Pizarro, Esther Fonseca Aguilar, Maria Magdalena Isac, K.V. Dhanapala, Kalyan Kumar Kameshwara, Ysrael Alberto Martínez Contreras, Geberew Tulu, José Fernando Mejía, Catalina Miranda, Shehe Abdalla Moh'd, Ricardo Morales Ulloa, K. Kayon Morgan, T. Lee Morgan, Sara Mori, Forti Ebenezah Nde, Silvia Panzavolta, Lluís Parcerisa, Carla Leticia Paz, Oscar Picardo, Carolina Piñeros, Pablo Rivera-Vargas, Alessia Rosa,  Lina Maria Saldarriaga, Adrián Silveira Aberastury, YM Tang, Kyoko Taniguchi, Ernesto Treviño, Carolina Valladares Celis, Cristóbal Villalobos, Dan Zhao  and Allison Zionts:** Data Collection and survey translation and adaptation.

## Declaration of Competing Interest

The authors declare that they have no known competing financial interests or personal relationships that could have appeared to influence the work reported in this paper.
